# Successful Renal Transplantation between Identical Twins with Very Brief Immunosuppression

**DOI:** 10.1155/2018/9842893

**Published:** 2018-06-27

**Authors:** Idris Yakubu, Abdolreza Haririan, Stephen Bartlett, Tracy Sparkes

**Affiliations:** ^1^Pharmacy, University of Maryland Medical Center, Baltimore, MD, USA; ^2^University of Maryland School of Medicine, Baltimore, MD, USA

## Abstract

Renal transplantation between monozygous identical twins provides an opportunity to utilize minimal immunosuppression to maintain stable allograft function, thereby alleviating the toxicities of immunosuppressive therapy. Despite monozygosity, there is a possibility of discordant protein presentation in identical twins that could trigger alloimmune response and lead to graft injury. Therefore, the optimal immunosuppression regimen in this patient population is unknown, and the safety of immunosuppression withdrawal remains controversial. Herein, we describe two patients who underwent successful renal transplantation from monozygotic identical twin donors. Monozygosity was determined using short tandem repeat (STR) analysis. All immunosuppression was successfully discontinued at 2 days and 3 weeks, respectively, after transplantation. Both patients are alive with functioning renal grafts at 1 year and 5 years after transplant, respectively. These two cases suggest that immunosuppression can be withdrawn safely and rapidly in select monozygous identical twin renal transplant recipients.

## 1. Introduction

Although advances in immunosuppression have led to improvement in short-term renal transplant graft outcomes, the vast majority of transplant recipients must remain on immunosuppressive medications for the lifetime of their allografts in order to prevent rejection. Due to the effects of immunosuppression, transplant patients are vulnerable to infection and malignancy. Additionally, immunosuppressive drugs increase the risk of posttransplant metabolic complications such as diabetes, hypertension, hyperlipidemia, and obesity, which are important risk factors for cardiovascular diseases—the leading cause of death in renal transplant recipients [1–5]. Living related kidney transplantation between identical twins provides a unique opportunity to further optimize patient and graft outcomes, given the low risk of allograft rejection. This low immunological risk also confers an opportunity to minimize or withdraw immunosuppression, thereby reducing or altogether avoiding the toxicities associated with immunosuppressive medications [6, 7].

This concept was demonstrated by the first successful kidney transplant that was performed over 6 decades ago by Dr. Joseph E. Murray at the Peter Bent Brigham Hospital in Boston. The recipient was a 24-year-old male who developed renal failure from glomerulonephritis and received a kidney transplant from his monozygotic identical twin brother. No immunosuppression was utilized, due to lack of availability. The recipient had a functioning graft for 9 years after transplant until he expired due to a myocardial infarction [7]. Since the discovery of pharmacological immunosuppression in the early 1960s and advances in understanding of the immunological mechanisms involved in rejection, various immunosuppression strategies have been used in this patient population [7–13]. When utilized, the appropriate choice of or the optimal dosing of the immunosuppressive agent(s) required remains unclear. Growing knowledge of the possibility of genetic differences between identical twins has further contributed to a variety of opinions on the optimal approach for these patients. Herein, we describe two cases of kidney transplantation between identical twins where immunosuppression was safely and rapidly withdrawn after transplant, despite the presence of donor specific antibody in one patient.

## 2. Case Presentation

### 2.1. Patient 1

**Figure 1:**
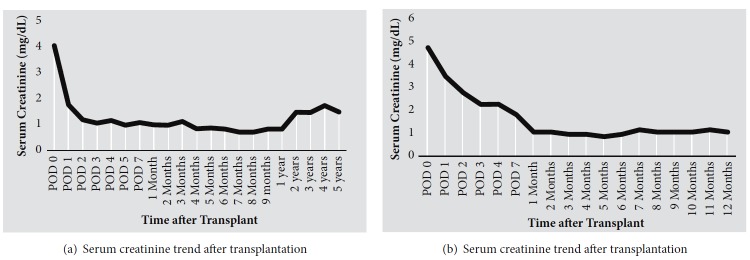


A 60-year-old Caucasian female with a history of ESRD secondary to hypertension and type 1 diabetes mellitus received a living related renal transplant from her HLA identical twin sister. At the time of transplant, monozygosity had yet to be determined. Therefore, she received a 3-day course of steroids, consisting of methylprednisolone 500 mg intraoperatively, followed by 250 mg and 125 mg on postoperative days (POD) 1 and 2, respectively. She also received maintenance immunosuppression of tacrolimus with a target trough level of 6 ng/mL and mycophenolic acid (Myfortic®) 360 mg twice daily. Postoperatively, blood samples from the donor and recipient were sent for short tandem repeat (STR) analysis in order to determine monozygosity. Given the use of maintenance immunosuppression, opportunistic infection prophylaxis was provided with oral sulfamethoxazole-trimethoprim 400-80 mg daily for* Pneumocystis jirovecii *pneumonia, fungal prophylaxis with oral clotrimazole 10 mg four times daily and cytomegalovirus (CMV) prophylaxis with valganciclovir 900 mg daily. She experienced immediate graft function and was discharged with no complications on postoperative day (POD) 5 with a serum creatinine (SCr) of approximately 1.0 mg/dL. The STR analysis occurred one week after transplant and demonstrated allelic identity at all 16 loci tested, consistent with monozygosity. Therefore, the transplant team decided to discontinue maintenance immunosuppression. Tacrolimus was stopped 12 days after transplant, and mycophenolate was stopped one week later. Infection prophylaxis was also discontinued once maintenance immunosuppression was stopped. She continues to have stable graft function at 5 years after transplant, with a baseline SCr of approximately 1.5 mg/dL (Figure 1(a)). She has experienced several episodes of mild acute kidney injury, which were attributed to her poorly controlled diabetes mellitus. She underwent a protocol allograft biopsy at 3 months after transplant, which showed no significant interstitial fibrosis and tubular atrophy, minimal arterial sclerosis, and mild arteriolar hyalinosis, with no evidence of acute allograft rejection. At 17 months after transplant, she underwent a second biopsy due to an elevation in SCr. The second biopsy showed mild tubular injury, without evidence of allograft rejection. No interventions were indicated based on the results of the 2 allograft biopsies, beside attempts to optimize her glycemic control. She has had no infectious or neoplastic complications from short-term immunosuppression.

### 2.2. Patient 2

A 71-year-old Caucasian female received a living related renal transplant from her identical twin sister. She developed ESRD due to chronic cystitis related to ureteral obstruction. Prior to transplantation, she made normal amounts of urine and had been on hemodialysis for 6 months. Her past surgical history included an ileal loop conduit urinary diversion and ileostomy drainage bag placement. Given the recipient's structural bladder anomaly, a ureteral anastomosis was made to the small bowel over a 6-French 30 cm double-J stent which extended from the renal pelvis out to the ileal conduit. Prior to transplant, monozygosity was confirmed via an STR analysis, which revealed that she was identical to her sister in all 16 polymorphic gene loci that were evaluated. Additionally, she was found to be a six-antigen match on HLA typing and had a negative anti-human globulin (AHG) crossmatch. Interestingly, a class II donor specific antibody (DSA) to DPB1*∗*05:01/DPA1*∗*02:02 with a mean fluorescence intensity (MFI) of 1,359 was identified four months prior to transplant. The antibody screening was repeated 10 days prior to transplant and revealed an MFI of 1,458. Based on the confirmation of monozygosity, the transplant team proceeded with transplantation without maintenance immunosuppression. She received a 3-day steroid taper course consisting of methylprednisolone 500 mg intraoperatively, followed by 250 mg and 125 mg intravenously on POD 1 and 2, respectively. She did not receive antibody induction therapy or further maintenance immunosuppression beyond the corticosteroids. Due to rapid immunosuppression withdrawal, she did not receive prophylaxis against opportunistic infections. She experienced immediate graft function, although her SCr was initially slow to decline (Figure 1(b)). Her hospital course was uncomplicated, and she was discharged home on POD 4 with a SCr of 2.3 mg/dL. She experienced a urinary tract infection (UTI) approximately 1 month after transplant, which resolved after ureteral stent removal and treatment with a 7-day course of oral ciprofloxacin. At 1 year after transplant, she continues to have excellent graft function with a baseline serum creatinine of 1.0 ng/mL (Figure 1(b)). A protocol kidney biopsy was attempted approximately 3 months after transplant, which however could not be performed due to lack of a clear window. Repeat posttransplant surveillance DSA screening with high resolution typing at the time of the attempted biopsy revealed persistence of Class II DPB1*∗*05:01/DPA1*∗*02:02 antibody, with a stable MFI of 1,030. She remains free from long-term complications due to brief immunosuppression, including infections, malignancy, or metabolic complications.

## 3. Discussion

The optimal immunosuppressive agent, magnitude, and duration of immunosuppression after monozygotic twin kidney transplantation remain controversial [9–13]. While our center's approach to date has been to rapidly minimize immunosuppression, this is not necessarily the approach followed elsewhere. In a study of 120 identical twin renal transplant recipients in the United States (US) and 12 recipients in the United Kingdom (UK), 68% and 33% of patients, respectively, were discharged on some form of immunosuppression, with steroids being the most commonly used agent in both groups [11]. At various follow-up time points, fewer patients were on each immunosuppressive agent. This suggests that, in some patients, immunosuppression was only given for a short period of time after transplantation. However, the timeframe of discontinuation of immunosuppression was not reported, and it is unclear if all cases were truly monozygotic identical twins. In the US cohort, although the 5-year graft survival rate among those who received immunosuppression was numerically better compared to those who did not, the difference was not statistically significant (93.9% versus 84.0%, *p* = 0.12) [11]. Additionally, in an Organ Procurement Transplant Network (OPTN) database study, Krishnan et al. evaluated the use of immunosuppressive agents at discharge and 6 months and 1, 2, and 3 years after transplant in identical twin kidney transplant recipients. The authors found that 71% and 34% of patients were on some form of immunosuppression at time of discharge and 1 year, respectively. Of those patients receiving immunosuppression at discharge, 59% were maintained on immunosuppression beyond 6 months after transplant and 33% of patients were on some form of immunosuppression therapy at 3 years after transplant. Patient and graft survival rates were similar from the time of transplant up to five years between those who received immunosuppression at discharge and those who did not (*p* = 0.98 and 0.54, respectively). After 5 years, patients who had received immunosuppression at discharge had worse patient survival rates, compared to those who did not (*p* ≤ 0.04), likely due to both short and long-term complications from immunosuppression. Renal function at 6 months onwards was superior in those not receiving immunosuppression (*p* ≤ 0.007). The average serum creatinine at 3 years after transplant was 1.46 ± 0.96 in patients receiving immunosuppression, compared to 1.13 ± 0.21 in those not on immunosuppression (*p* = 0.003). This difference in renal function was attributed to calcineurin inhibitor induced nephrotoxicity in patients receiving immunosuppression. The authors concluded that immunosuppression minimization in monozygotic twin transplantation may allow for better graft function and recommended the use of perioperative high dose steroids to minimize the potential for immune system activation from surgical tissue damage, followed by a rapid steroid taper to avoid the consequences of chronic immunosuppression [12].

The optimal method of determining monozygosity is unclear. A variety of methods have been used to identify monozygosity, including HLA typing, blood typing, chorionicity, evaluation of placenta after birth, deoxyribonucleic acid (DNA) finger printing, and reciprocal skin grafting [7, 14]. These methods have several limitations and are prone to misinterpretations. Standard HLA typing which is commonly used to determine zygosity may not accurately diagnose monozygosity, as 25% of dizygotic twins can be identical in all HLA loci [15, 16]. STR analysis has been shown to provide a greater sensitivity and precision in identifying differences between identical twins [17, 18]. Furthermore, advances in immunology and biology have shown that monozygous twins may not be completely identical as previously believed. Several mechanisms for differences, including postzygotic genetic changes, antenatal environmental effects, and postnatal environmental experiences have been postulated. These differences could lead to minor antigen presentation and trigger an alloimmune response against the transplanted organ [19, 20]. However, the possibility of antibody formation against donor HLA antigens in monozygous identical twins has not been reported in the literature. Our second patient was reported to have a class II antibody with borderline MFI, according to the threshold value used in our laboratory for detection of DSA. We are not certain if this was a false positive finding due to the limitations of MFI or the technique. One could postulate that the finding of antibody in this patient was possibly due to somatic genetic mutations in the donor that led to abnormal protein presentation and triggered antibody formation in the recipient.

In our center's twin transplant experience, the patients received a short course of steroids to decrease innate immune response resulting from ischemia-reperfusion injury, which has been shown to increase the risk of graft rejection [21, 22]. Previous studies have shown that the presence of preformed anti-HLA antibodies directed against a donor's class II antigens confers a high risk of humoral rejection [23, 24]. However, this does not necessarily correlate with an increased risk of rejection and graft loss in renal transplant recipients [25, 26]. Despite the presence of pretransplant DSA in patient 2, we decided not to use chronic immunosuppression, given her low antibody titers in the setting of a negative flow crossmatch and the results of HLA typing and STR. Therefore, we opted for minimum perioperative immunosuppression to prevent potential immunologic response to ischemia-reperfusion injury with close posttransplant surveillance and monitoring. Based on these experiences, our center performs STR analysis ahead of time in cases of identical twin transplants, to avoid unnecessary immunosuppression.

Previous published cases on identical twin transplantation have mostly been limited to a younger adult population with an average age of 30 years. Younger recipient age has been demonstrated to confer an increased risk of rejection after transplantation, due to a more robust immune response. Consequently, most transplant centers tend to favor more intense immunosuppression strategies in the younger transplant population [27]. The advanced age (>60 years old) of both patients provided an added opportunity to allow minimum immunosuppression utilization. Elderly patients have a less competent immune system, which reduces their risk of rejection after transplantation and contributes to a greater vulnerability to the toxicities of immunosuppressive therapy, including an increased risk of infections and death [28, 29].

In conclusion, these cases demonstrate that immunosuppression can be safely withdrawn in monozygotic identical twin transplant recipients. The consideration for immunosuppression withdrawal or minimization in this patient population needs to be individualized, and several important factors need to be considered, including confirmation of true monozygosity by means of reliable methods and analysis of the risk versus benefit of immunosuppression, including the likelihood of primary disease recurrence. We recommend the use of steroids perioperatively to prevent rejection resulting from activation of innate immune response due to ischemic reperfusion injury, along with close posttransplant monitoring.

## Abbreviations

ESRD: End stage renal disease
STR: Short tandem repeat
HLA: Human leukocyte antigen
CKD: Chronic kidney disease
AHG: Anti-human globulin
DSA: Donor specific antibody
MFI: Mean fluorescence intensity
IV: Intravenously
POD: Postoperative day
UTI: Urinary tract infection
US: United States
UK: United Kingdom
OPTN: Organ Procurement and Transplantation Network.
